# Identification of a New Splice Variant of the Human ABCC6 Transporter

**DOI:** 10.1155/2008/912478

**Published:** 2008-10-16

**Authors:** Maria Francesca Armentano, Angela Ostuni, Vittoria Infantino, Vito Iacobazzi, Maria Antonietta Castiglione Morelli, Faustino Bisaccia

**Affiliations:** ^1^Department of Chemistry, University of Basilicata, 85100 Potenza, Italy; ^2^Department of Pharmaco Biology, University of Bari, 75100 Bari, Italy

## Abstract

ABCC6 is a member of the adenosine triphosphate-binding cassette (ABC) gene subfamily C that encodes a protein (MRP6) involved in active transport of intracellular compounds to the extracellular environment. Mutations in ABCC6 cause pseudoxanthoma elasticum (PXE), an autosomal recessive disorder of the connective tissue characterized by progressive calcification of elastic structures in the skin, the eyes, and the cardiovascular system. MRP6 is codified by 31 exons and contains 1503 amino acids. In addition to a full-length transcript of ABCC6, we have identified an alternatively spliced variant of ABCC6 from a cDNA of human liver that lacks exons 19 and 24. The novel isoform was named ABCC6 Δ19Δ24. PCR analysis from cDNA of cell cultures of primary human hepatocites and embryonic kidney confirms the presence of the ABCC6Δ19Δ24 isoform. Western blot analysis of the embryonic kidney cells shows a band corresponding to the molecular weight of the truncated protein.

## 1. Introduction

ABCC6 belongs to the subfamily C of ATP-binding cassette (ABC) transmembrane transporters. The ABCC6 gene consists of 31 exons encoding for a protein of 1503 amino acids and has 17 transmembrane spanning domains and two conserved intracellular nucleotide binding domains (NBDs). ABCC6 is homologous (45% identity on amino acid level) to ABCC1, known to confer multidrug resistance to tumor cells [1]; for that reason, ABCC6 was classified as a multidrug resistance associated protein and also named MRP6. The NBDs contain two highly conserved Walker motifs critical for ATP binding and transmembrane transporter functions [2]. Mutations of the ABCC6 gene cause the pseudoxanthoma elasticum (PXE) (OMIM 177850 and 264800), amultisystemdisorder characterized by progressive calcification and degeneration of elastic fibers [3]. 

ABCC6 is highly expressed in human liver and to lesser extent in the proximal tubules of the kidney and only at very low levels, if at all, in tissues, such as skin, eyes, and cardiovascular system affected in pseudoxanthoma elasticum (PXE) [4, 5]. To date, genetic studies have identified 165 mutations, mainly missense and nonsense mutations, as well as large deletions (for a review see [6]). Since MRP6 is mainly expressed in liver and kidney, but only low levels are found in tissues affected by PXE, it has been suggested that PXE is primarily a metabolic disorder with secondary involvement of elastic fibers [7]. Despite the high correlation between ABCC6 mutations and PXE, the activity of MRP6 and its role in PXE remain largely unknown.

Recently, a splice variant leading to a 5 bp deletion in the ABCC6 transcript has been associated with cardiac dystrophic calcifications in mice [8].

In our study, we report the identification of a new variant of ABCC6 from human liver cDNA lacking exons 19 and 24. This splice variant was also confirmed in hepatic and renal cell cultures.

## 2. Materials and Methods

Human liver and kidney BD Marathon-Ready cDNA were purchased from Clontech. Primary human hepatocites (Cambrex) were maintained in culture medium (Cambrex) following the manufacture's instructions. Human embryonic kidney cells (Sigma) were maintained in high glucose Dulbecco's modified Eagle's medium (DMEM) containing 10% (v/v) fetal bovine serum, 2 mM L-glutamine, 100 U penicillin, and 100 *μ*g/mL streptomycin at 37°C in 5% CO_2_.

### 2.1. Cloning of cDNA Encoding Human ABCC6

To clone *ABCC6* cDNA, the forward primer 5′-CACCATGGCCGCGCCTGCTG-3′ and the reverse primer 5′-TCAGACCAGGCCTGACTCCTG-3′ were designed to cloning the blunt-end PCR product into pcDNA 3.1D/V5-His- TOPO expression vector (Invitrogen). PCR was performed using human liver cDNA and Platinum PCR SuperMix (Invitrogen). The PCR was carried out on a PTC-100 Peltier Thermal Cycler (MJ Research) and it consisted of 1 cycle of 95°C for 2 minutes, 30 cycles of 94°C for 45 seconds, 62°C for 1 minute, 68°C for 5 minutes and 30 seconds, and 68°C for 10 minutes. PCR product was isolated from agarose gel, purified with the MinElute Gel Extraction kit (Qiagen) and ligated into pcDNA.3.1D/V5-His-TOPO expression vector. The recombinant vector was transformed into TOP10 E. coli cells. Individual clones were cultured overnight in Luria Bertani broth with 100 *μ*g/mL ampicillin, and plasmid was isolated using the QIAprep Spin Miniprep kit (Qiagen).

### 2.2. RT-PCR Analysis

Total RNA was extracted from cultured cells using GenElute Mammalian Total RNAMiniprep Kit (Sigma). Before reverse transcription, the concentrations of total RNA were measured with the GeneQuant pro (Amersham International, Little Chalfont, UK) and RNA integrity was analyzed under UV light by visualization of 28S- and 18S-rRNA bands on a 1.5% agarose gel containing ethidium bromide. Total intact RNA (1 *μ*g) was reverse transcribed using GeneAmp RNA PCR Core Kit from Applied Biosystems with specific primers for the ABCC6 gene andMuLV reverse transcriptase, according to the manufacturers' instructions. Transcription reactions without the reverse transcriptase enzyme were performed for negative controls in subsequent PCR reactions.

To amplify region from exon 18 to exon 25 we used the following primers: 5′-GGCATGAATCTCTCCGGAG-3′ (forward primer in exon 18) and 5′-CTGGAGGGCAGCAGAGAC- 3′ (reverse primer in exon 25). The PCR was performed on human liver and kidney cDNA and cDNA of cell cultures. PCR consisted of 1 cycle of 95°C for 2 minutes; 30 cycles of 94°C for 45 seconds, 58°C for 1minute, 72°C for 2.5 minutes, and 72°C for 10 minutes. An aliquot of each amplicon was analyzed by ethidium bromide visualization on a 1.5% agarose gel to assess the size of the fragment, purified from the gel and directly sequenced.

### 2.3. Sequencing

The sequences of ABCC6 gene in the recombinant vector were verified using BigDye Terminator v3.1 Cycle Sequencing Kit (Applied Biosystems). The samples were analyzed with 3100 Avant Genetic Analyzer (Applied Biosystems) according to the manufacture's recommendation. The cDNA sequence of ABCC6 and its splicing variant ABCC6 Δ19Δ24 have been deposited to GenBank under IDs AM774324 and AM711638, respectively. The sequences of PCR products obtained by the amplification of exon18–exon25 region from cell cultures and from human liver and kidney cDNA have been performed by MWG Biotech.

### 2.4. Western Blotting

For immunoblot analysis of MRP6 expression, the whole cell lysate was isolated. HEK293 supplemented with protease inhibitors (0.1 mM PMSF, 5 *μ*g/mL aprotinin, 5 *μ*g/mL leupeptin, and 1 *μ*g/mL pepstatin) were centrifuged at 1200 rpm for 5 minutes. The pellet was resuspended in 1mL of ice cold RIPA buffer (PBS, Igepal CA-630 1%, sodium deoxycholate 0,5%, SDS 0.1%) and incubated on ice for 30 minutes; further disruption was obtained by sonication. After sonication, the lysate was incubated on ice for 30 minutes and then centrifuged at 10 000 ×g for 10 minutes at 4°C. The proteins were precipitated with five-volume of acetone at −20°C o/n, resuspended in Laemmli buffer and separated by SDS-PAGE (7%). Afterwards, the electrotransfer to an Immobilon-P transfer membrane (Millipore, Bedford, Mass., US) was confirmed by the reversible staining with Ponceau Red. After 20 minutes in incubation buffer (IB) (50 mM Tris, 150 mM NaCl, 0,5% Tween-20), membrane was incubated for 1 hour with a polyclonal human antibody, raised against amminoacids 1–70 of human MRP6 (Santa Cruz Biotechnology, Inc.), diluted 1:400 in IB. After three washings with washing buffer (WB) (50 mM Tris, 150 mMNaCl), the membrane was incubated with an horseradish peroxidase conjugated goat antirabbit antibody (Sigma immunochemical, St. Louis, Mo., USA), diluted 1:5000 in IB. Finally, the blot was washed three times with WB and the proteins were visualized with ECL (Immun-Star HRP, Biorad, Hercules, Calif., USA).

## 3. Results and Discussion

ABCC6 was amplified by PCR from human liver cDNA using specific primers and cloned into pCDNA3.1 vector. Surprisingly, the sequencing of some clones showed two different sequences of 4512 and 4137 nucleotides corresponding to the full length (ABCC6) and a shorter variant of ABCC6, respectively. A comparison of the shorter one with the exon/intron boundaries of ABCC6 gene revealed that the exons 19 and 24 were missing.

**Figure 1:**
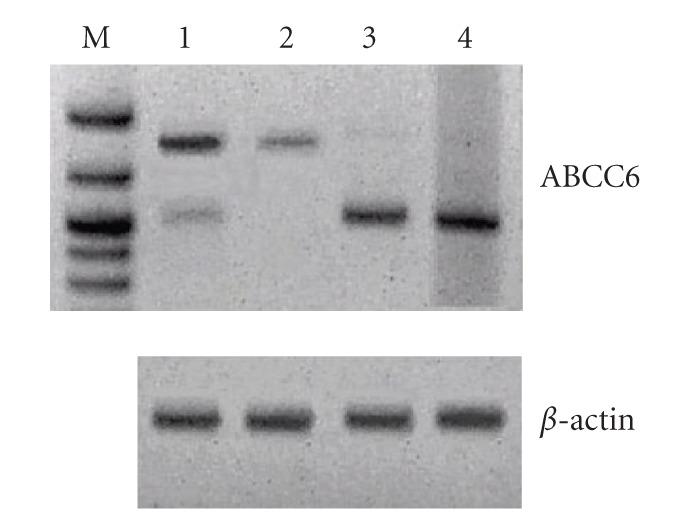
(Top) Expression pattern of exon18–exon25 region of ABCC6 from commercially available human liver and kidney cDNA (lanes 1 and 2), RNA reverse transcribed of primary human hepatocites (lane 3), and human embryonic kidney (lane 4). (Bottom) RT-PCR with *β*-actin primers as a control. The obtained PCR products were visualized by EtBr-stained agarose gel electrophoresis.

In order to verify if the short form, namely, ABCC6 Δ19Δ24, was a result of low-frequency splicing events or an ABCC6 variant, we amplified and sequenced the PCR products of the exon 18–25 region from cDNA of human liver, human kidney, primary human hepatocites (HI), and human embryonic kidney cells (HEK293). Liver and kidney showed essentially the complete exon 18–25 region (Figure 1 lanes 1 and 2), whereas the variant in which both exons are missing has been found mainly in HI and HEK293 (Figure 1 lanes 3 and 4). Then, we suggest that the isoform lacking exons 19 and 24 may be a product of a splicing variant differently distributed in various tissues and cell lines.

**Figure 2:**
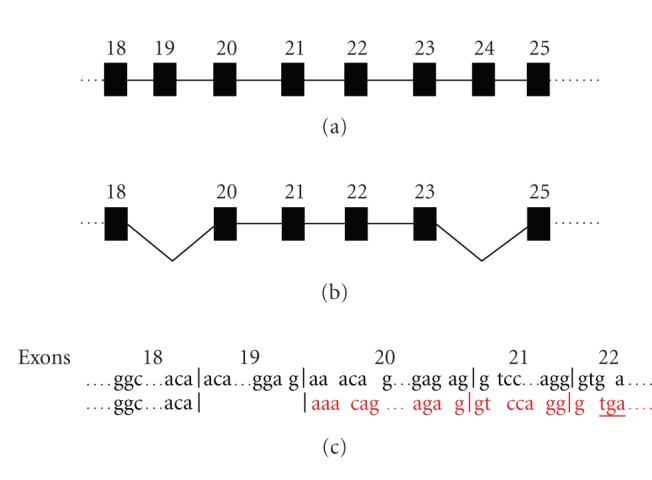
Splice event of the ABCC6 Δ19Δ24. (a) ABCC6 wild transcript; (b) splice event of exons 19 and 24; (c) shift of open reading frame as a consequence of splicing of exon 19 is indicated in red characters; a premature stop codon in exon 22 is underlined.

Deletion of the entire exon 19 causes a shift of the reading frame with insertion of a stop signal at nucleotides 2614–2616 (Figure 2). As consequence of the premature stop codon, the putative novel protein has a different and shorter C-terminus than that of native MRP6 protein. By a secondround nested PCR, aberrant splicing of the ABCC6 mRNA has been previously observed in tissues that do not express appreciable amount of the protein [5]. More recently, it has been demonstrated that a missense mutation in ABCC6 gene of mice creates a premature stop codon that, apart fromPXE, causes dystrophic cardiac calcification [8]. These findings suggest that truncated forms of ABCC6 can somehow affect cell activity.

For this reason, the identification of different splicing variants of ABCC6 in tissues such as liver and kidney, where ABCC6 is normally expressed, may be an important step in understanding the complex function of this gene and in clarifying the pathogenetic mechanisms of the correlated diseases.

**Figure 3:**
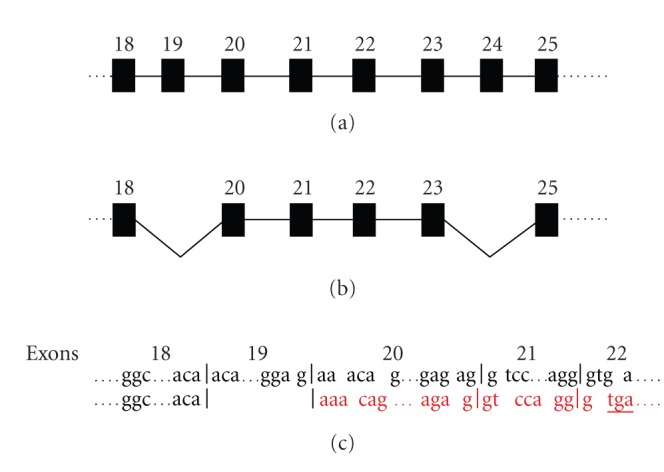
Western blot analysis of MRP6 in HEK293. The membrane probed with an MRP6 N-term specific antibody shows a clear band of about 100 kDa, corresponding to the translated protein from the ABCC6 Δ19Δ24 isoform (MRP6 Δ19Δ24).

To examine if the shorter variant encodes an expressed protein, we analyzed HEK293 by western blot analysis using an MRP6 N-terminus antibody as described in the methods (Figure 3). The predicted wild type protein of nearly 165 kDa (MRP6) and a more intense band of about 100 kDa corresponding to the truncated protein (MRP6 Δ19Δ24) are detected. The additional band between them could correspond to a different glycosylated form of the truncated protein. Western blot analysis is consistent with the expression level of the ABCC6Δ19Δ24 variant showed in Figure 1 and suggests that this isoform prevails in these cells.

Different speculative hypothesis on the function of the ABCC6Δ19Δ24 variant may be put forward.

The finding that ABCC6Δ19Δ24 variant codifies an expressed protein suggests a variety of functions for the ABCC6 gene and confirms that the alternative splicing is a diffuse mechanism to increase protein diversity in the ABC transporter superfamily.

We suggest that the translated protein of the ABCC6Δ-19Δ24 variant is a half transporter. In fact, translation of the nucleotide sequence of this variant yields a putative truncated protein of 871 amino acids, encompassing the first two transmembrane domains and the first NBD at the C-terminal end. It is well known that some other human ABC genes encode half transporters as a consequence of alternative splicing, such as the ABCA5 gene, which encodes a protein of 1642 amminoacids and a polypeptide of 925 amminoacids [9], and the human ABCB6 that produces two distinct molecular weight forms, localized in the outer mitochondrial membrane and in the plasma membrane [10].

## Abbreviations

ABC: Adenosine triphosphate-binding cassette
PXE: Pseudoxanthoma elasticum
ABCC6 Δ19Δ24: Adenosine triphosphate-binding cassette subfamily C member 6 lacking exons 19 and 24
NBD: Nucleotide binding domain
MRP: Multidrug resistance associated protein
HI: Primary human hepatocites
HEK293: Human embryonic kidney cells
rRNA: Ribosomal RNA
MuLV: Murine Leukemia Virus
EtBr: Ethidium bromide.
